# Primary care and community-based screening for Chagas disease in London, United Kingdom, August 2023 to January 2025

**DOI:** 10.2807/1560-7917.ES.2026.31.6.2500365

**Published:** 2026-02-12

**Authors:** Natalie Elkheir, Amel Alfulaij, Narayani Kathirgamakarthigeyan, Primary Care Delivery Team, UK Chagas Hub Volunteers, David Wingfield, Hannah G Davies, Jessica Carter, Debbie Nolder, Laura Nabarro, Peter L Chiodini, David AJ Moore, Primary Care Delivery Team, Silvia Gonzalez, Cristian Pico, Lukasz Radwanski, Antoinette McNulty, Marcelle Costa Marinho, Ashnee Dhondee, Zara Prem, Aldrich Menezes, Jasdeep Bhamrah, Martynas Stonkus, Wan Sze Shum, Monica Morim, Zuhaib Keekeebai, Alaliba Dokubo-Oguwuike, Desiree Fyle, Miguel Gonzalez Oquendo, Sobhash Jhuree, Yusuf Kabir, Maykon Melo, Moyosore Olukoga, UK Chagas Hub Volunteers, Nuria Sanchez Clemente, Ana García Mingo, Liliana Sanchez Zelaya, Laura Valderrama Penagos, Laura Lopez, Rodrigo Ville, Estefania Sienra-Iracheta, Temitope Fisayo, Pat Lok, Fionnuala Ryan, Ana Bayona, Jose Garcia Romero, Clare E. Warrell, Nicolas Massie, Cristina Fernandez-Turienzo, Valentina De Sario, Jenna Abaakouk, Karina Guzman Miranda, Cristina Suarez, Maria-Cristina Loader, Catherine Dominic, Marcela Bojorquez Segura, Heloise Gerber, Anna Bote-Casamitjana, Patricia Miralhes, Alyssa Chase-Vilchez, Amaya Bustinduy, Declan Crilly, Jaimie Wilson-Goldsmith, Barbara de Barros

**Affiliations:** 1 Clinical Research Department, London School of Hygiene & Tropical Medicine, London, United Kingdom; 2 Hospital for Tropical Diseases, University College London Hospitals, London, United Kingdom; 3 UK Chagas Hub (https://www.uclh.nhs.uk/uk-chagas-hub) University College London Hospitals, London, United Kingdom; 4 Hammersmith & Fulham Partnership Primary Care Network, London, United Kingdom; 5 Springfield Medical Centre, London, United Kingdom; 6 Members of the Primary Care Delivery Team and UK Chagas Hub Volunteers are listed under Collaborators; 7 Migrant Health Research Group, City St George's University of London, London, United Kingdom; 8 Diagnostic Parasitology Laboratory, London School of Hygiene & Tropical Medicine, London, United Kingdom; 9 Department of Clinical Parasitology, Health Services Laboratories, London, United Kingdom; * These authors contributed equally to this work and share second authorship.

**Keywords:** Chagas disease, Trypanosoma cruzi, screening, epidemiology, Latin America, primary care, community healthcare, migrant health

## Abstract

**BACKGROUND:**

Chagas disease (CD), a tropical parasitic disease caused by *Trypanosoma cruzi*, is increasingly recognised as a public health concern among Latin American migrants living in non-endemic settings. In London, United Kingdom (UK), home to over 160,000 people from endemic countries, no formal population-level CD screening programmes exist.

**AIM:**

To investigate the seroepidemiology of CD infection in Latin American adults living in London, UK.

**METHODS:**

A cross-sectional, observational study utilising questionnaires and serological (*T. cruzi*) screening was performed between August 2023 and January 2025. Adults born in South America, Central America or Mexico (or whose mothers were born there) were eligible to participate. Screening was offered in primary care sites and by point-of-care serological testing at community events. Outcomes were seroprevalence, screening yield, positive predictive value of screening tests and linkage to care.

**RESULTS:**

In primary care, of 2,739 eligible individuals offered screening, 276 (10.1%) accepted. At community events, 247 were screened. Of the 523 screened, 20 (3.8%) participants had positive screening tests, and CD was confirmed in 14 (2.7%), all born in Bolivia. Seroprevalence was lower in those screened in primary care (1.1%) than at community events (4.5%). The number needed to screen to detect one confirmed case (linked into care) was 37 overall, 92 in primary care and 22 at community events.

**CONCLUSIONS:**

Screening for CD through primary care in the UK is highly challenging, with both low uptake and low yield amongst those tested. Targeted community-based outreach approaches result in higher screening yield and linkage to care.

**Key public health message**

**What did you want to address in this study and why?**

We wanted to understand the prevalence of Chagas disease (CD), a neglected parasitic disease caused by *Trypanosoma cruzi*, among Latin American migrants in London, United Kingdom. Chagas disease can lead to heart disease in around one in three people, with onset 10–40 years after infection. We estimated screening population size, and measured uptake and reach of screening, when offered in primary care and at community events.

**What have we learnt from this study?**

Of ~500 Latin Americans screened in London between August 2023 and January 2025, 14 (2.7%) were confirmed to have *T. cruzi* infection. Prevalence was higher in people screened at community events vs primary care. In primary care, uptake was low but increased if clinicians directly contacted patients. Community screening was more effective in reaching more people, targeting high-risk groups, and linking people with CD to specialist care.

**What are the implications of your findings for public health?**

The identified seroprevalence of *T. cruzi* in London, supports the need for surveillance and targeted screening among Latin American migrants, particularly those born in Bolivia. Future work should focus on the real-world effectiveness of scaled up community-based screening initiatives, reducing barriers to accessing testing, and increasing awareness among the population at risk and healthcare providers.

## Introduction

Chagas disease (CD), caused by the parasite *Trypanosoma cruzi*, is endemic to the Americas [1], with an estimated seven million people with chronic infection globally [2,3]. While it has traditionally affected rural populations in Mexico, Central America and South America, CD is increasingly recognised as a public health concern in Europe and the United States because of migration from endemic regions [4-7]. Approximately one-third of individuals with chronic infection develop end-organ disease, most commonly affecting the heart, digestive system, or both, often decades after the initial infection. Detecting CD early and initiating antiparasitic treatment (with benznidazole or nifurtimox) may reduce the risk of progression to end-organ disease and almost eliminates the risk of vertical transmission [1]. Since most infections are asymptomatic, testing of individuals cannot be symptom-driven and case detection in non-endemic settings must be driven by screening strategies.

There were an estimated five million Latin American and Caribbean (LAC) migrants in Europe in 2020 [8]. This number has quadrupled since 1990, with high growth in recent years caused by economic hardship and political instability in several LAC countries [9]. By 2009, 4,290 cases of CD were registered among Latin American migrants in nine European countries, equating to an estimated prevalence of 1.3 cases per 1,000 migrants from endemic countries [10]. The highest numbers of reported cases in Europe are found, in order of prevalence, in Spain, Italy, the Netherlands, the United Kingdom (UK), Germany and France [11]. According to the Census 2021, more than 280,000 individuals residing in England and Wales were born in countries where CD is endemic, with over half living in London [12]. However, this figure underestimates the population at risk, as it does not account for second-generation migrants, i.e. those born in the UK to mothers from endemic regions who may be at risk of vertical transmission, or undocumented individuals, whose numbers are difficult to ascertain. Each year, ca 5,000 infants are born in the UK to women originating from CD-endemic countries [13]. 

Although *T. cruzi* serological testing and antiparasitic therapy are available in the UK, a 2016 estimate suggested that 97% of individuals with CD in the UK remained undiagnosed [14]. The UK Government’s Migrant Health Guide advises screening for migrants from endemic regions, particularly women of reproductive age and pregnant women [15]. Although blood donation screening for *T. cruzi* has been in place since 1998 [16], there is no formalised population screening strategy, nor surveillance for *T. cruzi*, in the UK.

In an earlier pilot study, we co-designed and implemented a community-based screening initiative for CD among Latin American migrants in London using point-of-care tests (POCTs) at community events [17]. Of 334 participants screened, 21% (n = 70) were confirmed to have *T. cruzi* infection, with a high rate of linkage to care. Based on this pilot, this study implemented and evaluated a primary care- and community-based screening initiative for CD in London between August 2023 and January 2025. The aims were to investigate the seroepidemiology of *T. cruzi,* as determined by different screening approaches, and to understand the barriers to and facilitators of successful screening pathways.

## Methods

### Study design and setting

We designed a cross-sectional, observational study in London, UK, with two arms: primary care and community settings, using questionnaires and serological screening tests, i.e. laboratory ELISA in primary care and POCTs at community events. Recruitment and screening took place between August 2023 and January 2025. This study is reported following STROBE guidelines for cross-sectional studies [18].

In the primary care arm, three sites (incorporating seven individual practices in total) in different parts of London recruited patients. The sites were selected based on both geography (aiming to reach a generalisable population of migrants living in different parts of London) and availability of local collaborating research partners.

In the community arm, pop-up stalls offering POCTs were set up at Latin American community events across London.

### Eligibility criteria

Adults (aged ≥ 18 years) born in, or whose mothers were born in, the 21 CD-endemic countries of Central America, South America and Mexico (Argentina, Belize, Bolivia, Brazil, Chile, Colombia, Costa Rica, Ecuador, El Salvador, French Guiana, Guatemala, Guyana, Honduras, Mexico, Nicaragua, Panama, Paraguay, Peru, Suriname, Uruguay, and Venezuela) were eligible to participate. There were no exclusion criteria other than age.

### Recruitment

#### Primary care

Eligible participants were systematically identified in primary care through searching electronic medical records for country of birth (21 CD-endemic countries) and/or ethnicity (Latin American) codes and/or main language spoken (Spanish or Portuguese). All eligible participants identified were sent a text message with study information and the option to respond to register interest. Interested participants were contacted by telephone to discuss the study and book an appointment for consent, questionnaire and a screening blood test. Practice staff also telephoned patients who did not respond to the text message to encourage participation. Appointments were offered during weekdays, weekday evenings and weekends, and a mixture of booked appointments and walk-in clinics (necessitating no appointment) were offered. Eligible participants were also identified and approached opportunistically by primary care staff at routine appointments. The study was also advertised through posters and videos on display in waiting rooms. Supplementary Figure S1 summarises the different ways participants were identified and tested in primary care.

#### Community

Stalls offering CD screening were set up at a series of Latin American community events, in partnership with local community groups and charitable organisations. These events were promoted through social media platforms and word-of-mouth. During each event, Spanish- and Portuguese-speaking healthcare professionals (members of the UK Chagas Hub, including doctors, nurses and midwives) explained the study to potentially eligible participants and obtained written informed consent from those who agreed to participate. These approaches (summarised in Supplementary Figure S2) were piloted, and findings of the pilot study have been published [17]. In contrast to most community events during the pilot, which were explicitly held for the purpose of providing CD POCTs (referred to as ‘CD-specific events’), in this study we took the opportunity to set up screening stalls at events that were organised by other community groups for unrelated purposes (such as an event on housing, and community celebrations on public holidays). All attendees at these events could approach the stall for information and take a CD POCT. Additionally, study healthcare professionals proactively walked around the events with leaflets to invite participation. This approach was intended to improve the generalisability of the study population, aiming to more closely reflect the wider Latin American community in London. No duplications were detected between those screened at community events and those from primary care.

### Data collection and sampling

Demographic details and CD risk factors, including age, sex, country of birth, maternal country of birth, family history of CD, and any prior testing or treatment, were collected through an online questionnaire (described in the pilot study [17]), administered via the Open Data Kit Collect application [19], and offered in English, Spanish and Portuguese.

Serological screening varied by setting. In primary care sites, venous blood was sampled and sent to the Hospital for Tropical Diseases (HTD clinical parasitology laboratory in London where the Wiener Chagatest recombinant v.4.0 enzyme-linked immunosorbent assay (ELISA) was performed. At community events (and in one primary care site which offered walk-in screening appointments), a finger-prick lateral flow POCT was used to detect IgG antibodies to *T. cruzi*. The Chembio Chagas STAT PAK Assay was performed in accordance with the manufacturer’s guidelines. 

### Participant follow-up

Participants with a positive test on either the ELISA or POCT were referred to a specialist CD clinic for confirmatory diagnostics, which included both the ELISA (if not already conducted) and an in-house indirect immunofluorescence antibody test (IFAT). In the case of discrepant (ELISA and IFAT) results, an immunoblot (Chagas Western Blot IgG assay, LDBio Diagnostics) was used. Two positive serological assays (ELISA, plus IFAT or immunoblot) were required to confirm the diagnosis of CD. The last case was followed up in May 2025.

Individuals with confirmed CD were offered comprehensive follow-up care, which included *T. cruzi* PCR testing at the diagnostic parasitology laboratory at the London School of Hygiene and Tropical Medicine, clinical assessment, a suite of cardiac investigations, gastrointestinal investigations if clinically indicated, antiparasitic therapy and family screening. Appointment reminders were sent via each participant’s preferred method of communication.

### Sample size calculation

The sample size calculation was based on meta-analysis of Latin American migrants screened in primary care and community settings in whom the pooled prevalence was 8.7%, (95% confidence interval (CI): 7.7–9.9) [20]. For this expected prevalence, applying a precision of 4.4%, i.e. half the prevalence estimate, and confidence of 95%, the sample size required was 193. Although these two groups were combined in the European meta-analysis, because of perceived differences between individuals who are recruited through primary care vs at community events in the UK, we did not believe it was valid to collapse the groups in our study and so the required sample size was 193 for each arm.

### Data analysis

Serological screening, followed by laboratory confirmation, was used to classify participants into a binary outcome of *T. cruzi* seropositive or seronegative. Seroprevalence was calculated overall, by setting and within specific risk groups stratified by age, sex and country of birth. The positive predictive value (PPV) of the screening method was determined by dividing the number of confirmed positive cases (in both ELISA and IFAT) by the total number of initial positive results. Screening yield, defined as the number of individuals that needed to be screened to identify one confirmed case, was calculated both in total and by setting.

Categorical variables were summarised using frequencies and percentages. For continuous variables, means and standard deviations (SD) were reported when data were normally distributed, while medians and interquartile ranges (IQRs) were used for non-normally distributed data. Prevalence estimates were presented with 95% CIs. Statistical comparisons of categorical variables were made using the chi-square test, and the Student’s t-test was applied to compare normally distributed continuous variables. A two-tailed p value of less than 0.05 was considered statistically significant. All analyses were conducted using STATA version 18 (StataCorp LLC).

## Results

### Identification of eligible participants 

In primary care, a search of electronic medical records identified 2,739 eligible participants (based on country of birth and ethnicity codes): 1,004 at Site 1, 625 at Site 2 and 1,110 at Site 3. Of those identified, 276 participants were recruited to screening in primary care between August 2023 and January 2025 (n = 83 in Site 1, n = 123 in Site 2 and n = 70 in Site 3) which equates to 10% uptake. Each site adopted a slightly different approach to implementing screening depending on local preferences and resources. All sites sent at least four text message invitations to all eligible participants and had posters on display. In Site 1, all patients were additionally telephoned at least once. In Sites 2 and 3, 50% of the patients were telephoned at least once. Following these efforts, in an attempt to enhance recruitment at Site 2, a study-specific walk-in clinic was offered (with POCT available on weekday evenings without appointments) to allow greater flexibility. This additional effort (an estimated 60 hours of a Spanish-speaking clinician’s time, 10 walk-in clinic days) boosted recruitment from 66 (10% of the total eligible) participants to 123 (20%) at this site. Supplementary Figure S3 outlines the approaches taken to recruit participants to screening in Site 2 as a case study, summarising the implementation outcomes and implications for primary care more broadly.

A total of 10 screening events were held between September 2023 and November 2024 in five boroughs of London with large Latin American populations (Southwark, Lambeth, Brent, Hackney and Camden), during which 247 individuals were screened. Three were CD-specific events (n = 92 screened, which was everyone who attended these events to the best of the authors’ knowledge) and seven were Latin American community events, of which four were Brazilian-focused (n = 155 screened).

### Participant characteristics

A total of 523 individuals were recruited to screening between August 2023 and January 2025 (Table 1). Of those screened, 339 (65%) were women, of whom 170 (50%) were women of reproductive age (aged 18–45 years). Participant characteristics differed by setting of recruitment, with more women and people born in Bolivia and Brazil recruited at community events compared with primary care. Most participants recruited in primary care reported having lived only in major urban cities, compared with those at community events who had lived in a more diverse mix of cities, towns and rural areas. Supplementary Figures S4 and S5 are maps of Latin America, visually representing locations where participants recruited to screening reported to have lived in the study questionnaires. Risk factors for CD also varied by screening setting. A higher proportion of participants recruited at community events had a first degree relative with CD (17% (42/247) vs 5% (14/276) in primary care) and reported previous testing for CD (9% (23/247) vs 5% (13/276) in primary care).

**Table 1:** Characteristics of study participants screened for Chagas disease in primary care and community settings, London, United Kingdom, August 2023–January 2025 (n = 523)

Characteristics	All		Primary care		Community events		p value^a^
	(n = 523)		(n = 276)		(n = 247)		
	n	%	n	%	n	%	
Mean age in years at screening (SD)	46 (12.5)		46.7 (12.8)		45.3 (12.1)		0.19
**Sex^b^**							
Males	182	34.8	112	40.6	70	28.3	< 0.05
Females	339	64.8	162	58.7	177	71.7	
Females aged 18–45 years	170	32.5	77	27.9	93	37.7	0.3
**Country of birth^c^**							
Argentina	11	2.1	9	3.3	< 5	< 2	< 0.001
Bolivia	77	14.7	16	5.8	61	24.7	
Brazil	155	29.6	52	18.8	103	41.7	
Chile	8	1.5	7	2.5	< 5	< 2	
Colombia	104	19.9	66	23.9	38	15.4	
Costa Rica	< 5	< 1	< 5	< 2	0	NA	
Ecuador	92	17.6	62	22.5	30	12.1	
Guatemala	< 5	< 1	0	NA	< 5	< 2	
Guyana	< 5	< 1	< 5	< 2	0	NA	
Honduras	< 5	< 1	< 5	< 2	0	NA	
Mexico	6	1.1	< 5	< 2	< 5	< 2	
Nicaragua	< 5	< 1	0	NA	< 5	< 2	
Paraguay	< 5	< 1	< 5	< 2	0	NA	
Peru	17	3.3	14	5.1	< 5	< 2	
Uruguay	< 5	< 1	< 5	< 2	0	NA	
Venezuela	25	4.8	25	9.1	0	NA	
Other	10	1.9	6	2.2	< 5	< 2	
**Self-reported risk factors for CD^d^**							
Mean years since last lived in a CD-endemic country (SD)**^e^**	20.3 (11.9)		20.1 (11.4)		20.5 (12.6)		0.67
Range in years	0–70		1–70		0–60		NA
**First-degree relative with CD**							
Yes	56	10.7	14	5.1	42	17.0	< 0.001
No	371	70.9	220	79.7	151	61.1	
Not known	96	18.4	42	15.2	54	21.9	
**Previous test for CD**							
Yes	36	6.9	13	4.7	23	9.3	< 0.05
No	454	86.8	250	90.6	204	82.6	
Not known	33	6.3	13	4.7	20	8.1	
**If tested, previous test result**							
Positive	8	1.5	1	0.4	7	2.8	0.2
Negative	23	4.4	9	3.3	14	5.7	
Unsure	5	1.0	3	1.1	2	0.8	
**If positive, previous treatment for CD received**							
Yes	4	0.8	1	0.4	3	1.2	0.56
No	3	0.6	0	NA	3	1.2	
Not known	1	0.2	NA		1	0.4	

CD: Chagas disease; NA: not applicable.

^a^ The p values refer to statistical tests assessing differences between participants recruited in primary care and those recruited at community events (t-test for mean age and chi-square test for all other categorical variables).

^b^ Two participants (both in primary care) did not disclose sex, answering ‘prefer not to say’ on the questionnaire.

^c^ Groups with fewer than five participants have been concealed (to < 5) to prevent identification.

^d^ For risk factors for CD, the ‘Not known’ category includes participants who did not answer that question, in addition to those who indicated they were unsure in the questionnaire.

^e^ The 21 endemic countries of South America, Central America and Mexico: Argentina, Belize, Bolivia, Brazil, Chile, Colombia, Costa Rica, Ecuador, El Salvador, French Guiana, Guatemala, Guyana, Honduras, Mexico, Nicaragua, Panama, Paraguay, Peru, Suriname, Uruguay and Venezuela.

Overall, the study population demography, as defined by country of origin, approximated that of the wider Latin American community of London (according to Census 2021) (Table 2). Of note, people born in Brazil were underrepresented in primary care (but not at community events where Brazilian communities were specifically targeted) and Bolivians were overrepresented overall, and in particular at community events.

**Table 2:** Comparison of study population (n = 523) with the Latin American population [12] in London, United Kingdom, August 2023–January 2025

Country of birth	Study population						London population (Census 2021 [12])	
	Primary care		Community events		Total			
	n	%	n	%	n	%	n	%
Brazil	52	18.8	103	41.7	155	29.6	55,790	37.0
Colombia	66	23.9	38	15.4	104	19.9	27,848	18.5
Ecuador	62	22.5	30	12.2	92	17.6	15,756	10.5
Guyana	< 5	< 2	0	0	< 5	< 1	10,717	7.1
Argentina	9	3.3	< 5	< 2	11	2.1	7,884	5.2
Venezuela	25	9.1	0	0.0	25	4.8	7,844	5.2
Bolivia	16	5.8	61	24.7	77	14.7	5,783	3.8
Peru	14	5.1	< 5	< 2	17	3.2	5,623	3.7
Mexico	< 5	< 2	< 5	< 2	6	1.2	5,096	3.4
Chile	7	2.5	< 5	< 2	8	1.5	3,480	2.3
Other	17	6.2	8	3.2	25	4.8	4,819	3.2

Groups with fewer than five participants have been concealed (to < 5) to prevent identification.

### Serological confirmation of Chagas disease

Of 523 participants, 20 (3.8%) had positive screening tests (four in primary care and 16 at community events) and an additional participant reported a previous positive test and was invited for further investigation and follow-up. 

Nineteen participants attended for confirmatory laboratory serology (90% of the 21 participants requiring follow-up); 18 had had a positive screening test, and one had screened negatively but reported a positive test previously. Of these 19, 14 (2.7% total population screened) were confirmed as true CD cases (two positive serological assays: ELISA/IFAT/immunoblot). In the one case of discordance between the confirmatory ELISA (weakly reactive) and IFAT (negative), the immunoblot was positive. All participants with confirmed CD were born in Bolivia.

True positive CD cases differed from those with a negative screening test by CD-risk factor profile, being older, more frequently reporting first-degree relatives with CD and previous testing for CD (Table 3).

**Table 3:** Risk factors for Chagas disease by serological screening result, London, United Kingdom, August 2023–January 2025 (n = 521^a^)

Characteristics	Negative (n = 507)		Confirmed cases (n = 14)		p value
	n	%	n	%	
Mean age, years (SD)	45.8 (12.5)		52.7 (10.4)		< 0.05
Mean years since last lived in Latin America (SD)	20.3 (11.9)		16.8 (8.6)		0.29
**Sex^b^**					
**Males**	178	35.1	4	28.6	1
Females	327	64.5	10	71.4	
Females aged 18–45 years^c^	166	50.8	4	40	0.45
First-degree relative with CD					
Yes	47	9.3	8	57.1	< 0.001
No	365	72	6	42.9	
Not known	95	18.7	0	0	
Previous test for CD					
Yes	24	4.7	11	78.6	< 0.001
No	450	88.8	3	21.4	
Not known	33	6.5	0	0	
If yes to above, previous test result					
Positive	0	0	8	57.1	< 0.001
Negative	22	4.3	1	7.1	
Not known	2	0.4	5	28.5	
If positive result reported above, previous treatment for CD					
Yes	NA	NA	4	50.0	NA
No	NA	NA	3	37.5	
Not known	NA	NA	1	12.5	

CD: Chagas disease.

^a^ Two participants with an initial positive screening test did not attend follow-up for confirmatory serology and have therefore been removed from this analysis.

^b^ Two participants (both identified in primary care, and both negative) did not disclose sex, answering ‘prefer not to say’ on the questionnaire.

^c^ Percentage is of total women.

For risk factors for CD, the ‘Not known’ category includes participants who did not answer that question or those who indicated they were unsure in the questionnaire.

The p values refer to statistical tests assessing differences between negative and confirmed cases (a t-test for the difference in mean age and chi-square test for all categorical variables).

Table 4 outlines the prevalence of Chagas disease in populations screened in this study, stratified by age, sex and country of birth.

**Table 4:** Prevalence of Chagas disease in populations tested through primary care and community screening in London, United Kingdom, 2023–2025 (n = 523)

Population	Confirmed cases	Population screened	Prevalence	
	n	n	%	95% CI
Total population screened	14	523	2.7	1.5–4.5
Screened in primary care	3	276	1.1	0.2–3.1
Screened at community event^a^	11	247	4.5	2.2–7.8
**Age-specific prevalence **				
18–29 years	0	61	0	0–5.9
30–39 years	3	117	2.6	0.5–7.3
40–49 years	3	143	2.1	0.4–6.0
≥ 50 years	8	202	4.0	1.7–7.7
**Sex^b^**				
Males	4	182	2.2	0.6–5.5
Females	10	339	2.9	1.4–5.4
Females aged 18–45 years	4	170	2.4	0.6–5.9

CI: confidence interval.

^a^ Ten of the 11 positive tests at were at CD-specific community events.

^b^ Two participants (both identified in primary care, and both negative) did not disclose sex, answering ‘prefer not to say’ on the questionnaire.

### Serological screening test characteristics

A total of 304 participants were screened with the Chagas POCT (247 at community events and 57 in primary care at Site 2 when a walk-in clinic was offered), and 219 with a laboratory ELISA. The overall PPV (of either screening test) was 78% (14 laboratory-confirmed positive tests of 18 people who had a positive screening test and attended for confirmatory testing). For the POCT alone, PPV was 79% (11 true positives/14), and for the laboratory ELISA it was 75% (3 true positives/4).

### Linkage to care

Of the 21 people requiring follow-up, 19 attended the tertiary hospital for confirmatory blood testing and specialist CD clinic appointment. Of the 14 true confirmed cases of CD detected through this study, all remained engaged in follow-up up to January 2025 (median 11 months of follow-up since screening).

### Screening yield

The number needed to screen to detect one confirmed case (linked into care) was 37 overall, 92 in primary care and 22 at community events. The number needed to screen to link one (previously untreated) person into care for antiparasitic therapy was 52. The number of women of reproductive age needed to screen to link one confirmed case into care was 43.

### Clinical assessment

After clinical assessment and baseline cardiac investigations (ECG, ambulatory ECG and echocardiogram) of the 14 cases of confirmed CD, 4 (29%) were *T. cruzi* PCR-positive and none were classified as having determinate disease, i.e. all cardiac investigations were normal, and no gastrointestinal symptoms were reported.

## Discussion

This study provides insights into the epidemiology of *T. cruzi* infection among Latin American populations in London, informing screening policy. *Trypanosoma cruzi* seroprevalence was higher among participants screened at community events compared with those in primary care, as was the screening yield. All participants confirmed to have CD were born in Bolivia, reflecting the high prevalence in some Bolivian regions.

Our finding of an overall seroprevalence of 2.7% among Latin American adults in London, United Kingdom, is lower than estimates from previous systematic reviews and meta-analyses of CD among Latin American migrants elsewhere in Europe, e.g. 4.2% in a review published in 2015, and 3.5% in an updated 2024 review [20,21]. The prevalence observed in our primary care setting (1.1%) is, however, broadly consistent with a modelled London-wide estimate published in 2016 (prevalence of 1.27% among Latin American migrants), and supports the finding of a lower prevalence compared with southern European countries with larger Bolivian populations [21]. Similar to other major European cities, the composition of London’s Latin American migrant population is very diverse, with a smaller proportion from countries with the highest endemicity of CD (such as Bolivia). Additionally, our study showed a predominance of migrants with origins in urban cities of Latin America where CD prevalence is generally low.

The much higher seroprevalence of 21% reported among participants in our earlier community screening pilot study likely reflected the targeting of the events at high-risk groups including many people with a previous CD diagnosis [17]. When restricting our analysis to only CD-specific community events, the seroprevalence was 11%. This present study demonstrates that prevalence is lower when people are attending community events for reasons other than specifically seeking a CD test, whereas targeted approaches enrich the screened population for people at higher risk and so generate a higher yield. Additionally, this study demonstrates that targeted community engagement and participatory research approaches can reach groups that did not initially attend community screening events, such as Brazilian populations who were not recruited in the pilot study. Although the community screening events conducted in this study had not been previously applied in the UK, similar approaches have been used elsewhere in Europe. For example, multiple approaches to community CD screening have been trialled in Barcelona, Spain, including hosting events at consulates and pharmacies [22,23]. Community events have also successfully reached high-prevalence groups for CD screening in France, Italy and Switzerland [24-26]. Additionally, initiatives in Switzerland have focused on reaching undocumented migrants and offering screening through primary care, where the CD seroprevalence in this particular group was 12.5% [27]. Our seroprevalence findings, and the difference in screening yield between primary care and community event settings, are consistent with studies performed in Spain. For example, the largest screening study in primary care in Spain reported a seroprevalence of 2.9% (of 766 participants) [28]. Whereas studies performed in the community in Spain have demonstrated a seroprevalence between 6 and 18% [22,29], noting some of these events were specifically targeting people from higher-endemicity countries such as Bolivia. Community based studies in Italy demonstrated a very similar seroprevalence to our analysis (restricted to CD-specific events) (of 8.7% and 9.5%, compared with ours of 11%) [25,30].

Although uptake in primary care was relatively low, this study demonstrated the acceptability and feasibility of the approaches used—such as walk-in clinics and targeted outreach, to reduce access barriers related to appointment scheduling. Furthermore, by offering screening in both primary care and community contexts, the study provides valuable insights into the populations that come forward under different circumstances. The difference in populations screened in primary care and at community events highlights the need for multifaceted outreach and screening strategies to maximise case detection, especially given that many affected individuals may not routinely access primary care services due to barriers such as lack of awareness, fear of migration status disclosure or limited-service provision [31].

Continued investment in community engagement, improved data quality in primary care, awareness and capacity building in primary care, and streamlined diagnostic pathways will be critical to enhancing CD surveillance and management in Europe and other non-endemic settings. Future research should explore methods to reach underrepresented groups, such as undocumented migrants. Qualitative and health systems research is also needed to optimise implementation, address barriers to uptake and strengthen linkage to care for affected individuals.

Some limitations of this study must be acknowledged. Firstly, it is important to consider that all confirmed cases of CD detected through screening were born in Bolivia, and so some findings (such as the number needed to screen to link one confirmed case into specialist care) should only be interpreted in this context, rather than generalised to the entire population eligible for screening. In primary care, identification of eligible participants relied on ethnicity, country of birth and language spoken codes, the latter two of which are not mandatory fields in UK electronic health records and may be incomplete or inconsistently recorded. This likely led to under-identification of the target population and may have biased the primary care sample. Secondly, children were not included in this study, and second-generation migrants were not accurately identified, limiting the generalisability of findings to these important subgroups. Finally, the use of convenience sampling at community events may have resulted in selection bias, as attendees may differ systematically from those who do not participate, although attempts to reduce this were made by offering screening predominantly at non-CD focussed community events, rather than CD-specific events, which were the focus of the pilot study.

## Conclusion

Screening for CD through primary care in the UK context is highly challenging, with both low uptake and low yield among those tested, with the exception of people born in Bolivia, in whom high risk of CD is confirmed by this study. Targeted community-based outreach approaches result in high screening yield and good linkage to care.

## Data Availability

The data that support the findings of this study are available from the corresponding author upon reasonable request.

## Supplementary Data

1356000 Supplement

## References

[1] de Sousa AS Vermeij D Ramos AN Jr Luquetti AO . Chagas disease. Lancet. 2024;403(10422):203-18. 10.1016/S0140-6736(23)01787-7 38071985

[2] Ribeiro ALP Machado Í Cousin E Perel P Demacq C Geissbühler Y The burden of Chagas disease in the contemporary World: The RAISE Study. Glob Heart. 2024;19(1):2. 10.5334/gh.1280 38222097 PMC10785959

[3] WHO . Chagas disease in Latin America: an epidemiological update based on 2010 estimates. Wkly Epidemiol Rec. 2015;90(6):33-43. 25671846

[4] Coura JR Viñas PA . Chagas disease: a new worldwide challenge. Nature. 2010;465(7301):S6-7. 10.1038/nature09221 20571554

[5] Coura JR Viñas PA Junqueira AC . Ecoepidemiology, short history and control of Chagas disease in the endemic countries and the new challenge for non-endemic countries. Mem Inst Oswaldo Cruz. 2014;109(7):856-62. 10.1590/0074-0276140236 25410988 PMC4296489

[6] Elkheir N Carter J García-Mingo A Chiodini P . Chagas disease in non-endemic settings. BMJ. 2021;373(901):n901. 10.1136/bmj.n901 33837041

[7] Elkheir N Moore DAJ . Rare in the country, not in the community: Chagas disease in the Latin American diaspora. Lancet Microbe. 2024;5(10):100982. 10.1016/j.lanmic.2024.100982 39305918

[8] Bayona-i-Carrasco J Avila-Tàpies R . Latin Americans and Caribbeans in Europe: A Cross-Country Analysis. Int Migr. 2020;58(1):198-218. 10.1111/imig.12565

[9] International Organization for Migration (IOM). World Migration Report 2024. Grand-Saconnex; IOM. [Accessed: 2 Oct 2025]. Available from: https://publications.iom.int/books/world-migration-report-2024

[10] Basile L Jansa JM Carlier Y Salamanca DD Angheben A Bartoloni A Chagas disease in European countries: the challenge of a surveillance system. Euro Surveill. 2011;16(37):19968. 10.2807/ese.16.37.19968-en 21944556

[11] European Centre for Disease Prevention and Control (ECDC). A comprehensive systematic review for identifying the risk factors for carrying a Trypanosoma cruzi infection in non-endemic countries. Stockholm: ECDC. [Accessed: 30 Apr 2025]. Available from: https://www.ecdc.europa.eu/en/publications-data/comprehensive-systematic-review-identifying-risk-factors-carrying-trypanosoma

[12] Office for National Statistics (ONS). Census 2021 (England and Wales). ONS; 2021. Available from https://www.ons.gov.uk/search?topics=9731,6646,3845,9497,4262,4128,7755,4994,6885,9724,7367&filter=datasets

[13] Office for National Statistics (ONS). Live births (England and Wales). ONS;2021. Available from: https://www.ons.gov.uk/peoplepopulationandcommunity/birthsdeathsandmarriages/livebirths/bulletins/birthsummarytablesenglandandwales/2021

[14] Requena-Méndez A Moore DA Subirà C Muñoz J . Addressing the neglect: Chagas disease in London, UK. Lancet Glob Health. 2016;4(4):e231-3. 10.1016/S2214-109X(16)00047-4 27013306

[15] UK Government Office for Health Improvement and Disparities (OHID). Chagas disease: migrant health guide. Advice and guidance on the health needs of migrant patients for healthcare practitioners. London: gov.uk; 2021. Available from: https://www.gov.uk/guidance/chagas-disease-migrant-health-guide

[16] The NHS Blood and Transplant (NHSBT) and the UK Health Security Agency. (UKHSA). Blood, tissue and organ donors: surveillance schemes. 2013. Available from: https://www.gov.uk/guidance/blood-tissue-and-organ-donors-surveillance-schemes

[17] Elkheir N Sanchez Zelaya L Valderrama Penagos L Lopez L Ville R Sienra-Iracheta E Community-based serological screening for Chagas disease in London: a cross-sectional, observational pilot study. BMJ Public Health. 2025;3(2):e002940. 41180358 10.1136/bmjph-2025-002940 PMC12574376

[18] von Elm E Altman DG Egger M Pocock SJ Gøtzsche PC Vandenbroucke JP The Strengthening the Reporting of Observational Studies in Epidemiology (STROBE) statement: guidelines for reporting observational studies. J Clin Epidemiol. 2008;61(4):344-9. 10.1016/j.jclinepi.2007.11.008 18313558

[19] Hartung C, Lerer A, Anokwa Y, Tseng C, Brunette W, Borriello G. Open data kit: tools to build information services for developing regions. Proceedings of the 4th ACM/IEEE International Conference on Information and Communication Technologies and Development; London, United Kingdom: Association for Computing Machinery; 2010. p. Article 18.

[20] Requena-Méndez A Aldasoro E de Lazzari E Sicuri E Brown M Moore DA Prevalence of Chagas disease in Latin-American migrants living in Europe: a systematic review and meta-analysis. PLoS Negl Trop Dis. 2015;9(2):e0003540. 10.1371/journal.pntd.0003540 25680190 PMC4332678

[21] Nepomuceno de Andrade G Bosch-Nicolau P Nascimento BR Martins-Melo FR Perel P Geissbühler Y Prevalence of Chagas disease among Latin American immigrants in non-endemic countries: an updated systematic review and meta-analysis. Lancet Reg Health Eur. 2024;46:101040. 10.1016/j.lanepe.2024.101040 39290806 PMC11407232

[22] Gómez I Prat J Essadek HO Esperalba J Serrat FZ Guiu IC Goterris L COVID-19: an opportunity of systematic integration for Chagas disease. Example of a community-based approach within the Bolivian population in Barcelona. BMC Infect Dis. 2022;22(1):298. 10.1186/s12879-022-07305-6 35346096 PMC8960226

[23] Silgado A Bosch-Nicolau P Sánchez-Montalvá A Cervià A Gomez-I-Prat J Bagaria G Opportunistic community screening of chronic Chagas disease using a rapid diagnosis test in pharmacies in Barcelona (Catalonia, Spain): study protocol and pilot phase results. Int J Public Health. 2022;67:1605386. 10.3389/ijph.2022.1605386 36531607 PMC9747760

[24] Lescure FX Paris L Elghouzzi MH Le Loup G Develoux M Touafek F [Experience of targeted screening of Chagas disease in Ile-de-France]. Bull Soc Pathol Exot. 2009;102(5):295-9. French. 20131423

[25] Pane S Giancola ML Piselli P Corpolongo A Repetto E Bellagamba R Serological evaluation for Chagas disease in migrants from Latin American countries resident in Rome, Italy. BMC Infect Dis. 2018;18(1):212. 10.1186/s12879-018-3118-5 29739357 PMC5941372

[26] Da Costa-Demaurex C Cárdenas MT Aparicio H Bodenmann P Genton B D’Acremont V . Screening strategy for Chagas disease in a non-endemic country (Switzerland): a prospective evaluation. Swiss Med Wkly. 2019;149(1314):w20050. 10.4414/smw.2019.20050 30946480

[27] Chappuis F Mauris A Holst M Albajar-Vinas P Jannin J Luquetti AO Validation of a rapid immunochromatographic assay for diagnosis of Trypanosoma cruzi infection among Latin-American Migrants in Geneva, Switzerland. J Clin Microbiol. 2010;48(8):2948-52. 10.1128/JCM.00774-10 20554821 PMC2916554

[28] Roca C Pinazo MJ López-Chejade P Bayó J Posada E López-Solana J Chagas disease among the Latin American adult population attending in a primary care center in Barcelona, Spain. PLoS Negl Trop Dis. 2011;5(4):e1135. 10.1371/journal.pntd.0001135 21572511 PMC3082512

[29] Ramos JM Ponce Y Gallegos I Flóres-Chávez M Cañavate C Gutiérrez F . Trypanosoma cruzi infection in Elche (Spain): comparison of the seroprevalence in immigrants from Paraguay and Bolivia. Pathog Glob Health. 2012;106(2):102-6. 10.1179/2047773212Y.0000000013 22943545 PMC4001495

[30] Antinori S Galimberti L Grande R Bianco R Oreni L Traversi L Chagas disease knocks on our door: a cross-sectional study among Latin American immigrants in Milan, Italy. Clin Microbiol Infect. 2018;24(12):1340.e1-6. 10.1016/j.cmi.2018.03.017 29555394

[31] Ciftci Y Blane DN . Improving GP registration and access for migrant health. Br J Gen Pract. 2022;72(715):56-7. 10.3399/bjgp22X718301 35091400 PMC8813121

