# Supplementary material for: Primary care and community-based screening for Chagas disease in London, United Kingdom, August 2023 to January 2025
Source: Euro Surveill. 2026 Feb 12;31(6):2500365. doi: 10.2807/1560-7917.ES.2026.31.6.2500365 (PMC12905528; doi:10.2807/1560-7917.ES.2026.31.6.2500365)
Supplement: Supplement [file 25-00365_ELKHEIR_Supplement.pdf]

Supplementary material

This supplementary material is hosted by Eurosurveillance as supporting information alongside the article "*Primary care and community-based screening for Chagas disease in London*" on behalf of the authors, who remain responsible for the accuracy and appropriateness of the content. The same standards for ethics, copyright, attributions and permissions as for the article apply. Supplements are not edited by Eurosurveillance and the journal is not responsible for the maintenance of any links or email addresses provided therein.

Figure S1. Participant recruitment flowchart in primary care

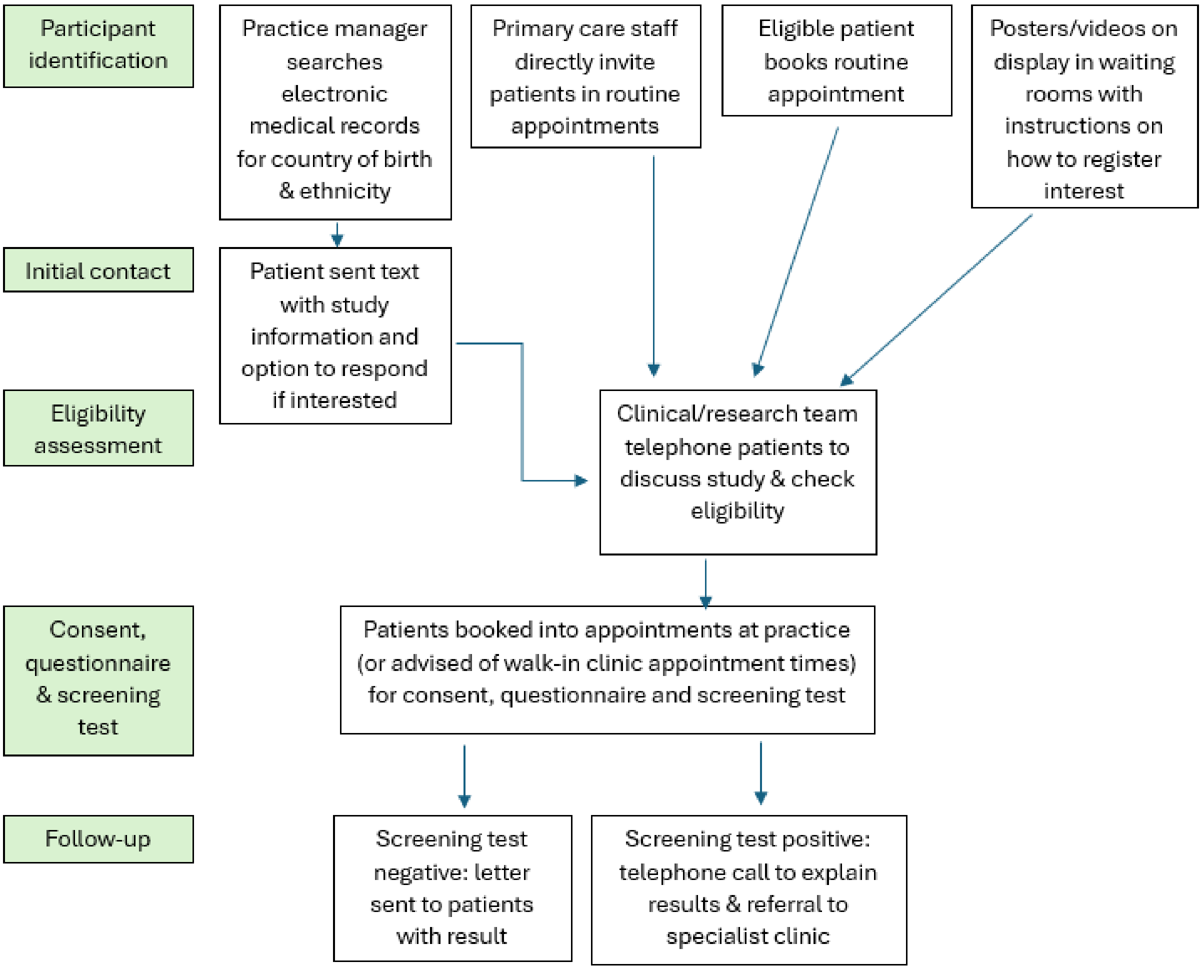

Figure S2. Participant recruitment flowchart at community events

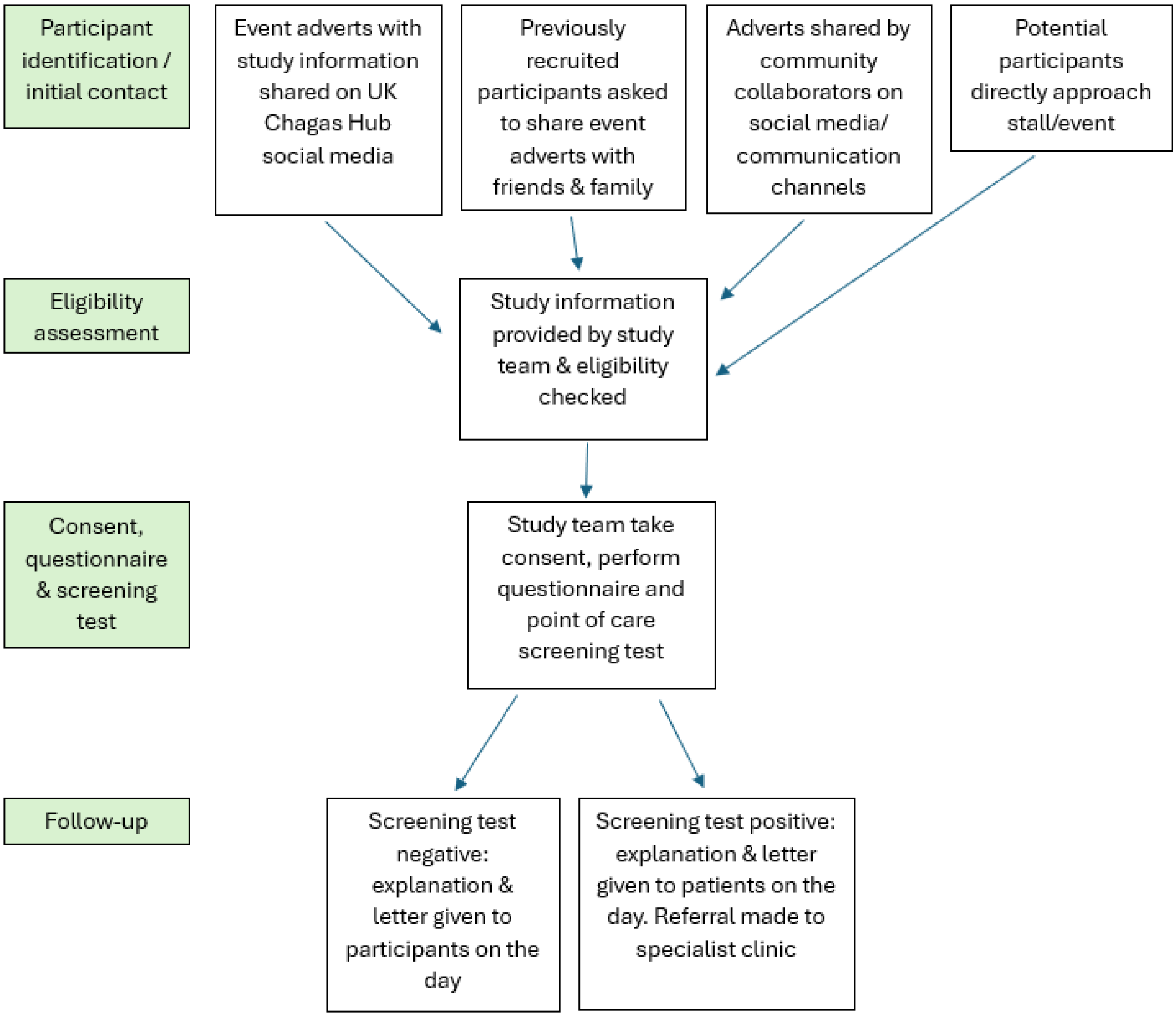

Figure S3. Case study of one primary care site's experience of implementing screening for Chagas disease

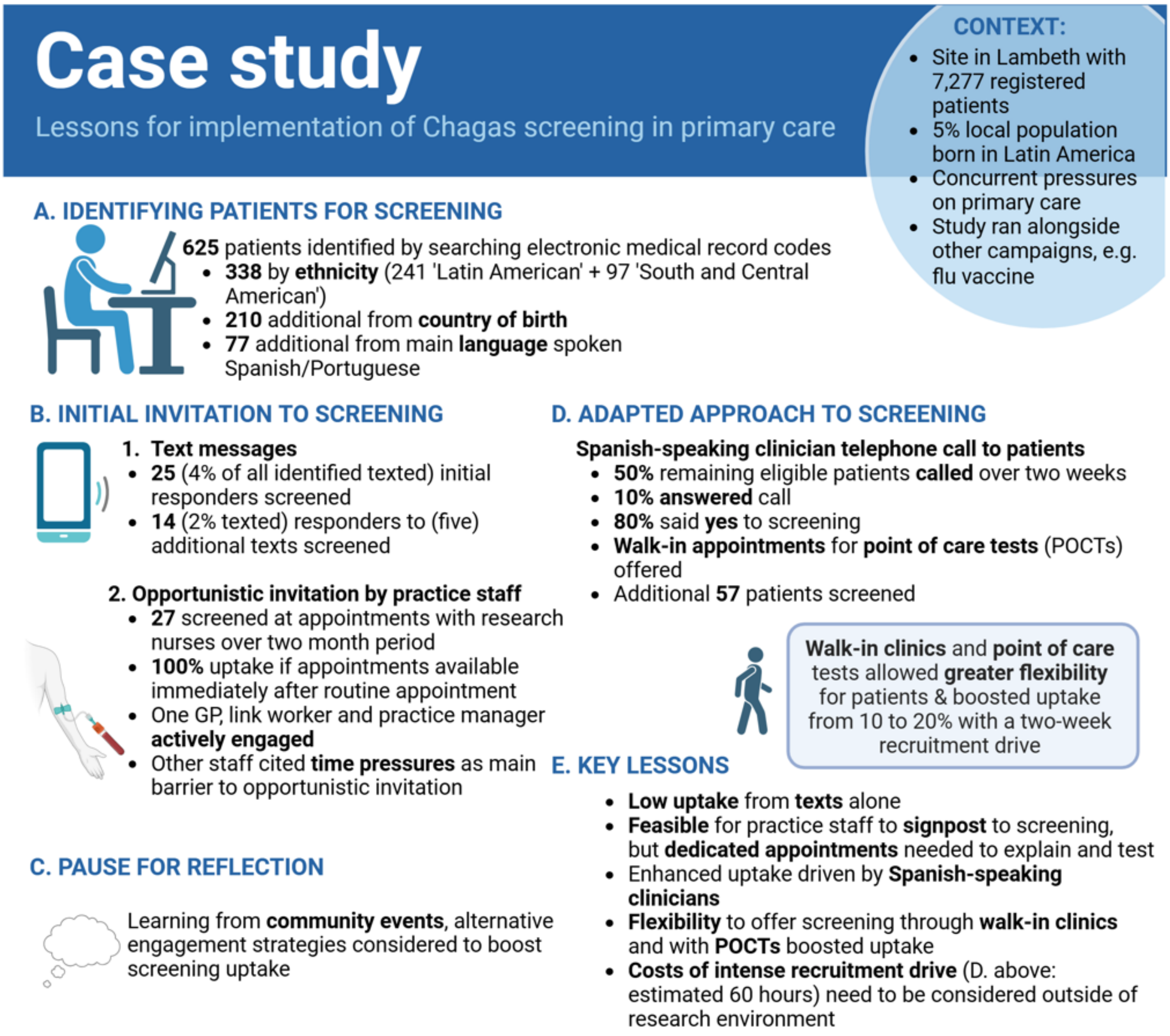

Figure S4. Locations in Latin America where participants recruited to screening in primary care had lived (self-reported in questionnaire)

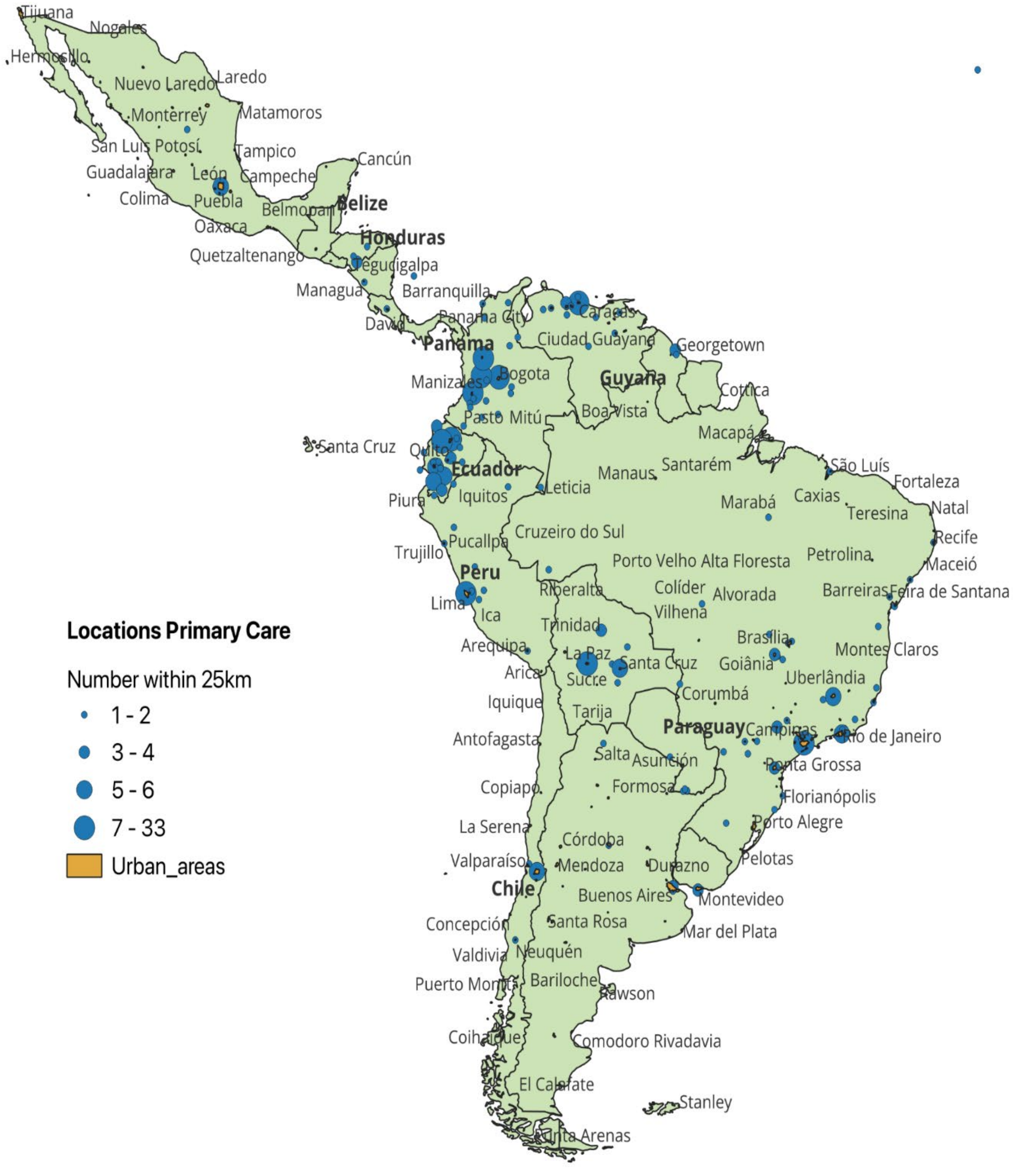

Participants were asked to select up to three locations on a digital interactive map (coordinates) where they had lived in Mexico, Central America or South America. To ease interpretation, coordinates within 25km of each other have been clustered, so larger blue circles indicate that more participants selected that location. This map was generated in QGIS software and is freely distributable under a GNU General Public License. Urban areas (from Natural Earth Data) are overlayed in orange

Figure S5. Locations in Latin America where participants recruited to screening at community events had lived (self-reported in questionnaire)

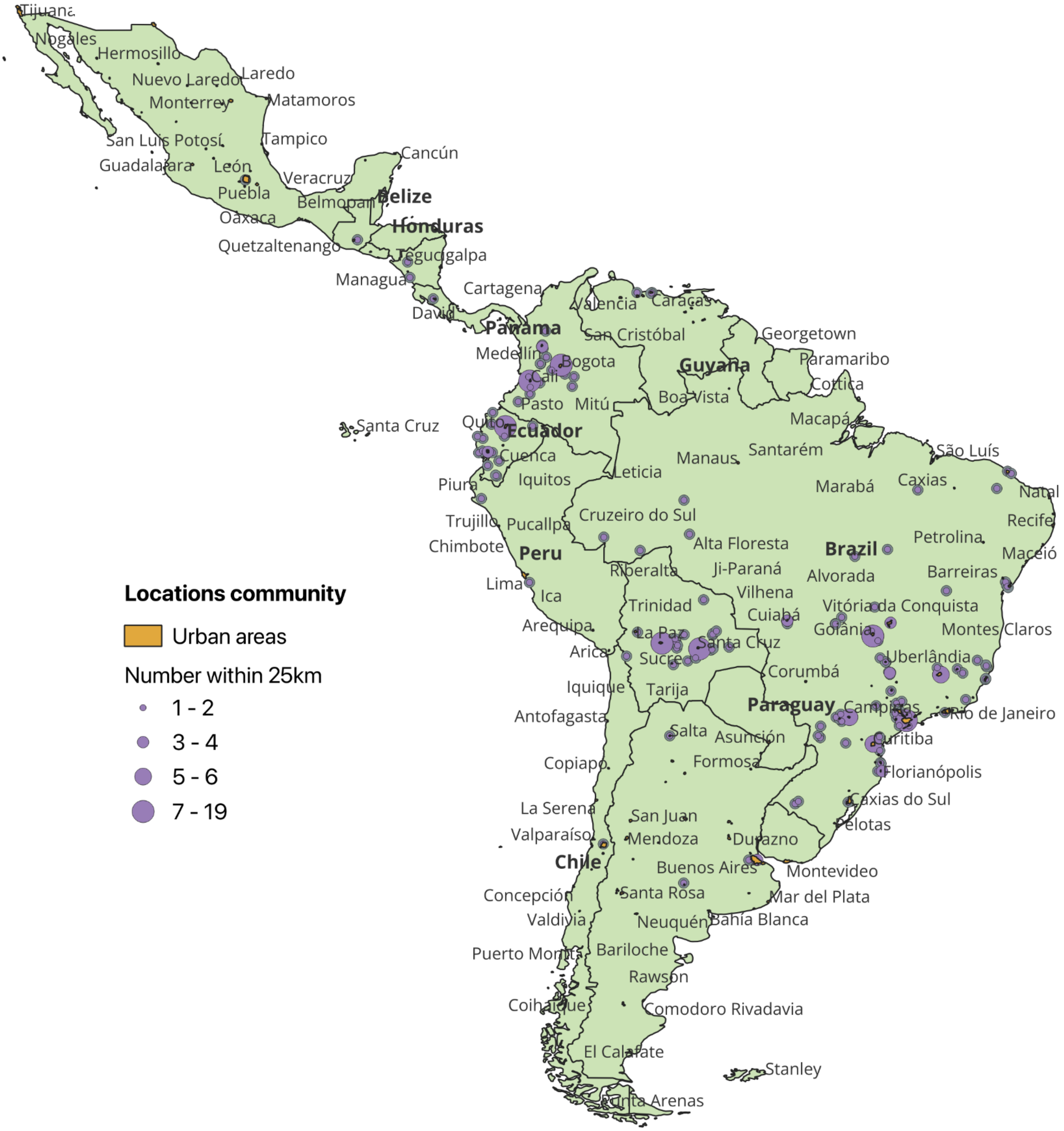

Participants were asked to select up to three locations on a digital interactive map (coordinates) where they had lived in Mexico, Central America or South America. To ease interpretation, coordinates within 25km of each other have been clustered, so larger purple circles indicate that more participants selected that location. This map was generated in QGIS software and is freely distributable under a GNU General Public License. Urban areas (from Natural Earth Data) are overlayed in orange.
